# Repetitive pulsed-wave ultrasound stimulation suppresses neural activity by modulating ambient GABA levels via effects on astrocytes

**DOI:** 10.3389/fncel.2024.1361242

**Published:** 2024-03-27

**Authors:** Tatsuya Mishima, Kenta Komano, Marie Tabaru, Takefumi Kofuji, Ayako Saito, Yoshikazu Ugawa, Yasuo Terao

**Affiliations:** ^1^Department of Medical Physiology, Kyorin University School of Medicine, Mitaka, Japan; ^2^Institute of Innovative Research, Tokyo Institute of Technology, Yokohama, Japan; ^3^Radioisotope Laboratory, Kyorin University School of Medicine, Mitaka, Japan; ^4^Department of Human Neurophysiology, School of Medicine, Fukushima Medical University, Fukushima, Japan

**Keywords:** ultrasound neuromodulation, astrocyte, TRPA1, ambient GABA, network activity

## Abstract

Ultrasound is highly biopermeable and can non-invasively penetrate deep into the brain. Stimulation with patterned low-intensity ultrasound can induce sustained inhibition of neural activity in humans and animals, with potential implications for research and therapeutics. Although mechanosensitive channels are involved, the cellular and molecular mechanisms underlying neuromodulation by ultrasound remain unknown. To investigate the mechanism of action of ultrasound stimulation, we studied the effects of two types of patterned ultrasound on synaptic transmission and neural network activity using whole-cell recordings in primary cultured hippocampal cells. Single-shot pulsed-wave (PW) or continuous-wave (CW) ultrasound had no effect on neural activity. By contrast, although repetitive CW stimulation also had no effect, repetitive PW stimulation persistently reduced spontaneous recurrent burst firing. This inhibitory effect was dependent on extrasynaptic—but not synaptic—GABA_A_ receptors, and the effect was abolished under astrocyte-free conditions. Pharmacological activation of astrocytic TRPA1 channels mimicked the effects of ultrasound by increasing the tonic GABA_A_ current induced by ambient GABA. Pharmacological blockade of TRPA1 channels abolished the inhibitory effect of ultrasound. These findings suggest that the repetitive PW low-intensity ultrasound used in our study does not have a direct effect on neural function but instead exerts its sustained neuromodulatory effect through modulation of ambient GABA levels via channels with characteristics of TRPA1, which is expressed in astrocytes.

## Introduction

Transcranial ultrasound stimulation (TUS) is a new non-invasive neuromodulation technique that can penetrate the skull to influence biological tissue. TUS has higher spatial resolution than transcranial magnetic (TMS) (De Deng et al., 2013) or electrical direct current (tDCS) (Reinhart et al., 2015) stimulation, which are already in practical use. It can stimulate a focused area only a few millimeters in diameter in the deep brain (Legon et al., 2014). Depending on the stimulation site and parameters, excitatory (Lee et al., 2016; Yu et al., 2016) and inhibitory (Dallapiazza et al., 2018; Nakajima et al., 2022) effects have been observed in human subjects and animals. For example, stimulation of the motor cortex in animals induces electromyographic (EMG) signals in the corresponding forelimb, hindlimb, or tail muscles, indicating that TUS can induce action potentials (Oh et al., 2019; Duque et al., 2022). TUS also improved cognitive functions such as learning and memory (Eguchi et al., 2018) and ameliorated depressive-like behaviors (Wang et al., 2023). Inhibitory effects of TUS include long-term attenuation of synaptic transmission (Niu et al., 2022), reduction of somatosensory input at the thalamus (Dallapiazza et al., 2018), and suppression of epileptiform electroencephalography (EEG) discharges and seizure episodes (Min et al., 2011; Hakimova et al., 2015). These opposing effects on neural activity are thought to be due to differences in stimulation site, intensity, ultrasound frequency, and other parameters (Dell’Italia et al., 2022; Blackmore et al., 2023). However, no consistent relationships have yet been identified.

To elucidate these relationships, we need to clarify the mechanisms underlying ultrasound stimulation. Ultrasound produces both thermal and mechanical effects at the target location. However, the temperature increase is negligible at low intensities (Tufail et al., 2010), and the acoustic radiation force and vibration exert their mechanical effects without causing cavitation (Yoo et al., 2022). Biophysically, mechanosensitive channels are responsive to the mechanical effects of ultrasound (Blackmore et al., 2023). Various mechanosensitive channels are expressed in the brain, with different expression patterns depending on cell type and region. Most are cation channels permeable to calcium ions, and activation causes cell depolarization and an increase in intracellular calcium levels. Techniques that investigate the mechanism of action of ultrasound at the cellular level are based predominantly on calcium imaging, a powerful technique that allows us to observe two-dimensional intracellular calcium levels. However, calcium imaging fails to provide details on how changes in calcium levels affect neuronal function. To tackle this relationship between calcium levels and neural function, electrophysiological analysis using whole-cell recording is essential, but because ultrasound stimulation is incompatible with whole-cell recording (Tyler et al., 2008; Prieto et al., 2018), this approach has yet to be implemented. Furthermore, most previous studies of the effect of ultrasound on cellular activity used cells expressing exogenous channel genes (Cadoni et al., 2023; Xu et al., 2023; Zhu et al., 2023); verifying the effects of ultrasound on naïve, wild-type neurons expressing only endogenous channels would provide important insights. In this study, to elucidate the effects of ultrasound on neural function and the underlying mechanism of action, we made whole-cell recordings from wild-type hippocampal neurons cultured in networks on astrocytes by optimizing the conditions of ultrasound stimulation.

## Materials and methods

### Ultrasound stimulation setup and transducer characterization

This study used a 2-mm diameter, 5-MHz center frequency, immersion-type planar transducer (5C1I, KGK, Japan) for the ultrasound stimulation. The transducer was submerged in a recording chamber and placed under the objective lens at an angle of elevation of 35°. The ultrasound stimulation protocols were as follows. Pulsed wave: 0.5-ms tone-burst duration, 100-Hz pulse repetition frequency, 5% duty cycle, and total sonication duration of 2 s; this stimulation pattern has been reported to inhibit epileptiform EEG discharges and seizure episodes in model animals (Min et al., 2011; Chen et al., 2020). Continuous wave: 100-ms duration; this has been reported to have excitatory effects (Yu et al., 2021; Dell’Italia et al., 2022).

The transducer was driven by 5 MHz electrical sinusoidal waveforms produced by an electronic stimulator (SEN-3301, Nihon Kohden, Japan) and function generator (33500B, Keysight, United States) and amplified by a 50 W RF power amplifier (T142-4749A, Thamway, Japan). The power amplifier was connected to a custom-made matching circuit (Garcia-Rodriguez et al., 2010) connected to the transducer. All systems were connected to each other with 50-Ω coaxial cables. The needle hydrophone (NH0200, Precision Acoustics, United Kingdom) was mounted on a 3-D positioner to calibrate the transducer and measure the pressure profile. After locating the center of the transducer, 2-D raster scans in both the XY and XZ planes were acquired in degassed water. The spatial-peak pulse-average intensity (I_SPPA_) and spatial-peak temporal average intensity (I_SPTA_) were calculated from pressure signals per industry standards (NEMA, 2004).

### Primary neuronal culture

All experimental procedures were performed in accordance with the Guidelines for the Care and Use of Laboratory Animals approved by Kyorin University (Reference number 222). C57Bl/6 J mice were purchased from CREA Japan (Tokyo, Japan), and primary neuronal cultures were prepared from the hippocampus of postnatal day 0 mice, following a previously described protocol (Mishima et al., 2014). Neonatal pups were briefly anesthetized by hypothermia and decapitated. Neurons from the hippocampus of the neonatal pups were dissociated with trypsin (5 mg/mL at 37°C for 10 min), triturated with a siliconized pipette, and plated at a density of 3–4 × 10^4^/cm^2^ on a glial feeder layer that was prepared approximately 1 week before the neuronal culture preparation. Under conditions without astrocytes, hippocampal neurons from E17 embryos were used to prevent astrocyte contamination. Immunostaining confirmed that the number of astrocytes was less than 1% of the total cell count (Supplementary Figure S1). Neurons were cultured at 37°C in a humidified incubator with 95% air, 5% CO_2_ in DMEM containing 2% B-27 supplement and 2 microM Ara-C, and used at 14–21 days *in vitro*. The present study used glial cells obtained from the hippocampus in all cases. To obtain the glial cells, the hippocampus from newborn mouse pups was dissociated and triturated with a siliconized pipette. Cells were plated in T-75 tissue-culture flasks and maintained in DMEM containing 10% fetal bovine serum for 7 days. At 7 days, the flasks were shaken at 150 rpm in an environmental shaker to remove oligodendrocytes, microglia, and neurons (Levison and McCarthy, 1991). To facilitate cell adherence, the remaining attached cells were removed with trypsin and plated onto 12-mm glass coverslips coated with polyethylenimine. Most (90–95%) of these cells were astrocytes, as determined by staining with the polyclonal antibody against glial fibrillary acidic protein (Sigma, United States). Each group of data was obtained from at least 3 different batches of cultures.

### Electrophysiology

For whole-cell recordings, borosilicate glass electrodes with filaments (external diameter of 1.5 mm; inner diameter of 1.17 mm; Warner Instruments, United States) were pulled on a Flaming/Brown micropipette puller (P-97, Sutter Instruments) to form micropipettes with a resistance of 5–7 MΩ. Recordings were made in an external solution containing the following (mM): 135 NaCl, 4.0 KCl, 1.6 CaCl_2_, 0.8 MgCl_2_, 10 HEPES, and 10 glucose, adjusted to pH 7.4 with NaOH; osmolarity was 290 mOsm/kg. To maintain cells under physiological conditions (35 ± 0.5°C), the external solution was heated by an in-line heater (DTC-300, Dia Medical, Japan) and a thermoplate (TP-110NLR, Tokai Hit, Japan). The external solution was continuously perfused at a flow rate of 1.5 mL/min at all times during the experiment to prevent changes in osmotic pressure due to evaporation. Temperature was monitored by a thermistor placed in the recording chamber with a volume of 1.5 mL. Miniature excitatory (mEPSCs) and inhibitory (mIPSCs) postsynaptic currents and tonic GABA_A_ currents were recorded by an EPC-9 (HEKA, Germany) in the whole-cell voltage-clamp configuration at a holding potential of −70 mV, as described previously (Mishima et al., 2021). For mEPSC recordings, the internal solution was composed of the following (in mM): 115 K-gluconate, 10 KCl, 15 phosphocreatine, 10 HEPES, 4.0 MgCl_2_, 1.0 EGTA, 0.1 CaCl_2_, 2.0 MgATP, and 0.2 NaGTP, adjusted to pH 7.3 with KOH. mEPSCs were recorded in the external solution supplemented with 1 μM tetrodotoxin (TTX), 10 μM bicuculline, and 50 μM D-AP5 to block fast sodium spikes, GABAergic inhibitory postsynaptic currents, and NMDA-mediated currents. For mIPSC and tonic GABA_A_ current recordings, the internal solution was composed of the following (in mM): 97 KCl, 40 CsCl, 10 HEPES, 4.0 MgCl_2_, 1.0 EGTA, 0.1 CaCl_2_, 2.0 MgATP, and 0.2 NaGTP, adjusted to pH 7.3 with KOH. mIPSCs were recorded in the external solution supplemented with 1 μM tetrodotoxin (TTX), 10 μM CNQX, and 50 μM D-AP5 to block fast sodium spikes and glutamatergic excitatory postsynaptic currents.

Whole-cell current-clamp recordings were conducted to analyze network activity. The internal solution was composed of the following (in mM): 115 K-gluconate, 10 KCl, 15 phosphocreatine, 10 HEPES, 4.0 MgCl_2_, 1.0 EGTA, 0.1 CaCl_2_, 2.0 MgATP, and 0.2 NaGTP, adjusted to pH 7.3 with KOH. Recorded cells had a membrane potential of at least −60 mV. The recordings were discarded if ultrasound stimulation caused a steep change in resting membrane potential more positive than −50 mV.

A miniature-event analysis was performed in MiniAnalysis software 6.0.7 (Synaptosoft, United States). The frequency distribution of events was calculated by kernel density estimation (KDE) using a uniform function with a window width of 500 ms. For single-shot ultrasound, event frequencies were normalized by the mean probability function for the 5 s before stimulation. For repeated ultrasound stimulation, relative spike frequencies were normalized by the average spike frequency 5 min before the repeated stimulation.

The tonic GABA_A_ current was defined as the difference between the baseline holding current (*I_hold_*) and *I_hold_* in the presence of 10 μM bicuculline (Nusser and Mody, 2002). Bicuculline-containing solution was delivered to the recorded neuron immediately after the end of 3 min of repeated PW or after 5 min of AITC administration by an injection system consisting of a glass capillary positioned close to the soma of the recorded cell. *I_hold_* was calculated by generating an all-points histogram from 1-s segments before and during the application of 10 μM bicuculline. A Gaussian distribution was fitted to the histogram or single Gaussians were fitted to the positive side of the all-points histogram if the Gaussian distribution was skewed to the left when high-frequency mIPSCs were recorded. The peak of the fitted Gaussian distribution was used to assess the mean *I_hold_*. Series resistance was monitored continuously throughout all experiments by measuring the capacitive current response to a 5-mV voltage step. Series resistance ranged from 8 to 25 MΩ and was not compensated in any of the experiments.

### *In vitro* pharmacology

For inhibition of inhibitory synaptic transmission, we used the GABA_A_ receptor antagonists bicuculline (Oh et al., 2019) (10 μM; Enzo Life Sciences # BML-EA149) and SR95531 (Semyanov et al., 2003) (500 nM, Gabazine; ABCAM # ab120042). For activation of TRPA1, we used AITC (Shigetomi et al., 2012) (100 μM in DMSO, allyl isothiocyanate; Nacalai # 01415–92). For inhibition of TRPA1, we used HC-030031 (Paumier et al., 2022) (40 μM; Alomone # H-105). The final concentration of DMSO in the external solution was 0.01% or lower for all groups, and this was also used as the vehicle control.

### Statistical analysis

Statistical analyses were performed in Igor Pro 8.0 (WaveMetrics, United States) and EZR (Saitama Medical Center, Jichi Medical University, Saitama, Japan) (Kanda, 2013), which is a graphical user interface for R (The R Foundation for Statistical Computing, Austria). All statistical tests in this study were two-tailed non-parametric tests, conducted after assessing normality with the Shapiro–Wilk test. Differences were considered significant at *p* < 0.05. Single-variable comparisons were made with the Mann–Whitney *U*, Wilcoxon signed-rank, and Friedman tests. Group comparisons were made with the Steel test. In box-plot graphs, the box extends from the 25th to 75th percentiles, the whiskers range from the lowest to the highest values, and the line in the box corresponds to the median.

## Results

### Single-shot ultrasound stimulation has no effects on neural activity

**Figure 1:**
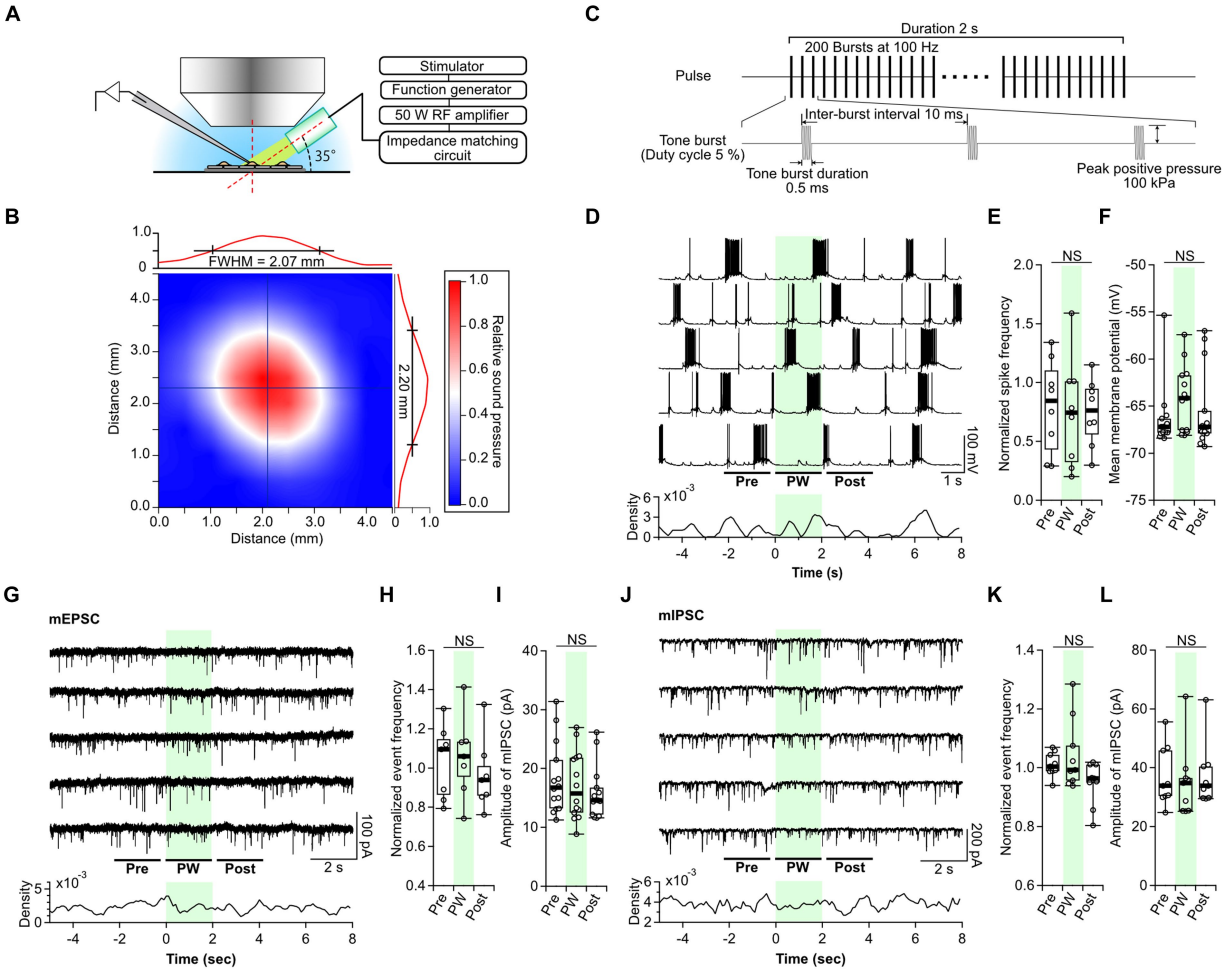
Single-shot pulsed-wave stimulation has no effect on neural activity. **(A)** Schematic showing whole-cell recording and stimulation of cells with the ultrasound transducer. **(B)** The color map shows the two-dimensional sound pressure distribution at 3 mm from the transducer. **(C)** Schematic of the pulse-wave (PW) stimulation. **(D)** Representative trace of network activity and event frequency before (pre), during (PW), and after (post) single-shot PW stimulation (green shading). **(E)** Comparison of the average spike frequency calculated from the 2-s pre, PW, and post intervals indicated by the bars in **(D)** (*n* = 8 independent experiments, two-tailed Friedman test, *p* = 0.4169). **(F)** Comparison of the mean membrane potentials calculated from the 2-s pre, PW, and post-intervals shown in **(D)** (*n* = 13 independent experiments, two-tailed Friedman test, *p* = 0.06271). **(G)** Representative mEPSC traces and event frequency before, during, and after single-shot PW stimulation. **(H)** Comparison of the average event frequency calculated from the 2-s pre, PW, and post intervals indicated by the bars in **(G)** (*n* = 7 independent experiments, two-tailed Friedman test, *p* = 0.8669). **(I)** Comparison of average event amplitude calculated from the 2-s pre, PW, and post intervals shown in **(G)** (*n* = 13 independent experiments, two-tailed Friedman test, *p* = 0.5004). **(J)** Representative mIPSC traces and event frequency before, during, and after single-shot PW stimulation. **(K)** Comparison of average event frequency calculated from the 2-s pre, PW, and post intervals indicated by the bars in **(J)** (*n* = 9 independent experiments, two-tailed Friedman test, *p* = 0.2359). **(L)** Comparison of the average event amplitude calculated from the 2-s pre, PW, and post intervals indicated by the bars in **(J)** (*n* = 9 independent experiments, two-tailed Friedman test, *p* = 0.7165). Light green shading indicates the stimulus period. NS, not significant.

In this study, we investigated the effects of ultrasound on neural network activity and synaptic transmission to clarify the impact of ultrasound on brain function. Primary cultures of mouse hippocampal neurons were grown on astrocytes, and the effects of ultrasound were analyzed via whole-cell patch-clamp recordings. The neuronal cultures were placed under a microscope, and a 5-MHz, 2-mm-diameter transducer was placed between the objective lens and the neuron (Figure 1A). The distance between the transducer and the recorded neuron was 3.5 to 4.5 mm, and the maximum sound pressure was 150 kPa (Supplementary Figure S2). The diameter of the focal area measured by the full-width at half-maximum was 2.1 to 2.2 mm (Figure 1B). Thus, the ultrasound was delivered over the entire field of view. Ultrasound experimental results appear to depend on the stimulation pattern (Yu et al., 2021; Niu et al., 2022); therefore, two types of single-shot stimulation patterns were used in this study. The first was a single-shot pulsed wave (PW) with 0.5-ms tone burst duration (TBD) and 5-MHz fundamental frequency, presented 200 times at a pulse repetition frequency (PRF) of 100 Hz (Figure 1C). The total sonication time was 2 s, with a duty cycle (DC) of 5%. The pulse intensity integral (*PII*) was calculated to be 0.192 J/cm^2^, with a pulse average intensity (I_SPPA_) of 0.38 W/cm^2^ and a temporal average intensity (I_SPTA_) of 19.2 mW/cm^2^. Stimulation patterns with low DC and PRF, including the pattern used here, have been reported to inhibit epileptiform EEG discharges and seizure episodes in model animals (Min et al., 2011; Chen et al., 2020). The second was a single-shot continuous wave (CW) with a frequency of 5 MHz, presented for 100 ms (Oh et al., 2019; Duque et al., 2022). The I_SPPA_ was 0.7 W/cm^2^. Because high-DC stimulation patterns have been reported to have excitatory effects (Yu et al., 2021), we used a continuous wave with a 100% DC pattern. The duration of the CW was designed to be the same as the total duration of the TBDs for the PW.

As reported in previous papers, the gigaohm seals required for patch-clamp often broke when ultrasound stimulation was applied during whole-cell recording (Tyler et al., 2008; Prieto et al., 2020; Duque et al., 2022). Furthermore, the cells themselves were sometimes ruptured by the ultrasound. Cell rupture occurred only in the recorded neuron and not in the surrounding cells. Observed expansion of cells suggests that the negative pressure produced by ultrasound forced the internal solution within the electrode to flow out into the cell, resulting in cell rupture. These phenomena occurred more frequently under higher sound pressure conditions, and stable recordings were possible at positive pressures up to 100 kPa. Because of this, all ultrasound stimulation was performed at 100 kPa in this experiment.

First, we examined the neural responses to single-shot PW stimulation. In culture, primary neurons form synapses with surrounding neurons at random and, once matured (after day 14), show synchronized recurrent burst firing (Verstraelen et al., 2014). Therefore, we recorded network activity from neurons at 14–21 days *in vitro*. While recording spontaneous network activity, a 2-s PW was applied five times at 20-s intervals (Figure 1D). A comparison of the frequency of action potentials and membrane potentials before, during, and after stimulation showed no effect of PW stimulation (Figures 1E,F). Next, we analyzed the effects of PW stimulation on excitatory synaptic transmission (Figure 1G). We compared the frequency and amplitude of AMPA-mediated mEPSCs before, during, and after stimulation and again found no effect of PW stimulation (Figures 1H,I). Similarly, we analyzed inhibitory synaptic transmission (Figure 1J) and found that ultrasound stimulation had no effect on the frequency or amplitude of GABA-mediated mIPSCs before, during, or after stimulation (Figures 1K,L).

We next examined the impact of single-shot CW stimulation on neurotransmission. We found no changes in the frequency of action potentials or the membrane potential before and after CW stimulation (Supplementary Figure S3). There was also no effect of CW stimulation on the frequency or amplitude of mEPSCs or mIPSCs (Supplementary Figures S4, S5). These results indicate that neither single-shot PW stimulation nor single-shot CW stimulation affects neural function at a sound pressure of 100 kPa.

### Repetitive pulsed-wave stimulation decreases spontaneous neural network activity in a GABAergic-dependent manner

**Figure 2:**
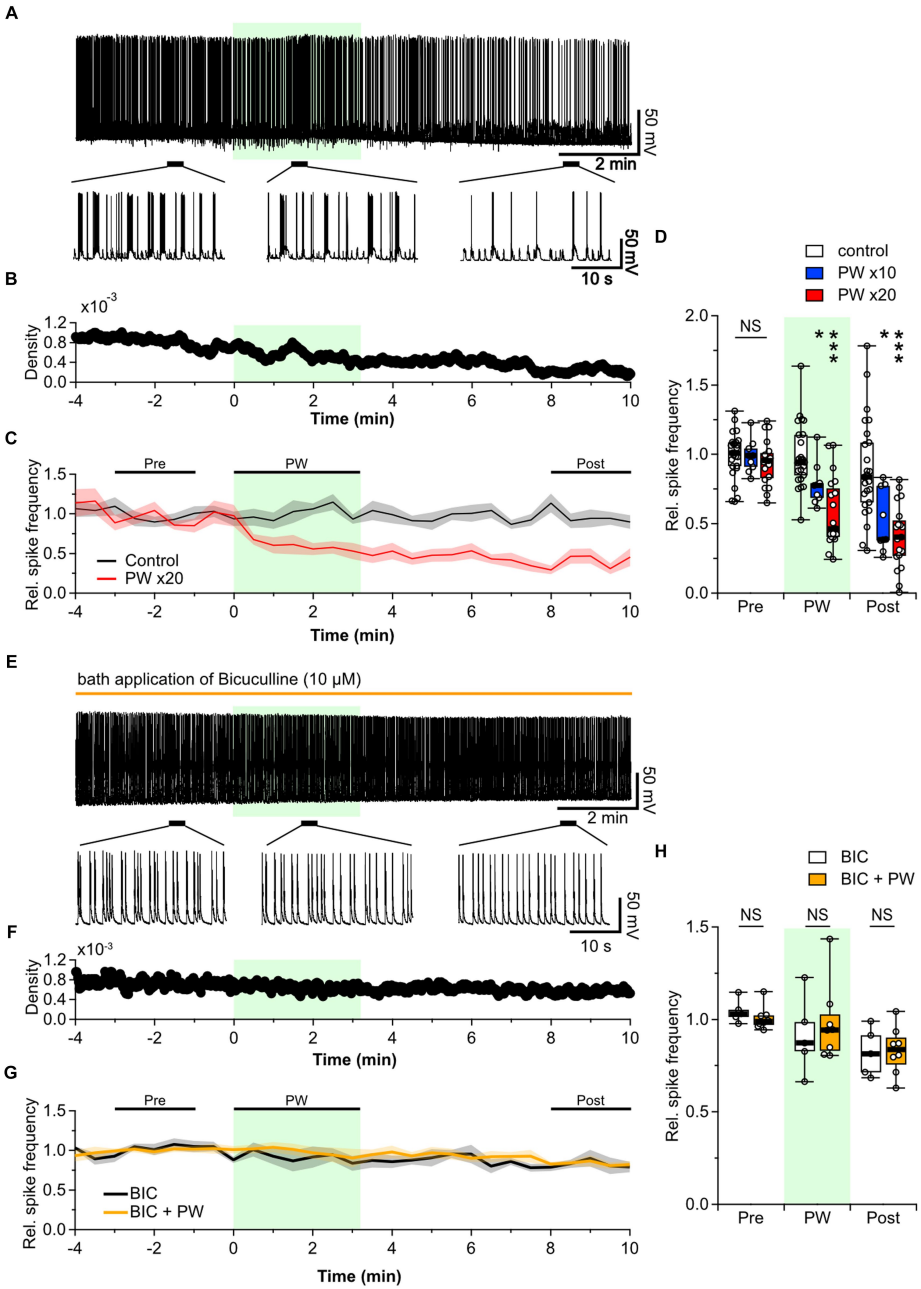
Repetitive PW stimulation inhibits neural activity. **(A)** Representative trace of network activity before, during, and after repetitive PW stimulation (upper). Magnified traces of the indicated regions (bottom). **(B)** Spike frequency of the action potentials shown in **(A)**. **(C)** Average spike frequency in the pre, PW, and post intervals. **(D)** Comparison of the average spike frequency calculated for the 2-min pre and post intervals and for the 3-min PW stimulation period, as indicated by the bars in **(C)** (control *n* = 25 independent experiments; x10 PW *n* = 9 independent experiments; x20 PW *n* = 19 independent experiments, two-tailed Steel test). **(E)** Representative trace of network activity before, during, and after repetitive PW stimulation in the presence of bicuculline (BIC) (upper). Magnified traces of the indicated regions (bottom). **(F)** Spike frequency from **(E)**. **(G)** Average spike frequency before, during and after repetitive PW stimulation in the presence of bicuculline. **(H)** Comparison of the average spike frequency calculated for the 2-min pre and post intervals and the 3 min PW interval, as indicated by the bars in **(G)** (control *n* = 5, bicuculline *n* = 8 independent experiments, two-tailed Mann–Whitney *U* test). Light green shading indicates the stimulus period. The mean trace is indicated by a solid line, with the SEM shaded. ^*^p < 0.05, ^***^p < 0.001. NS, not significant.

Since no effect was seen with single-shot stimulation, we investigated whether any effect could be observed when the PW stimulation was repeated for extended periods. We used the PW stimulation described above but repeated 20 times at 10-s intervals, for a total stimulus duration of approximately 3 min (192 s). This repetitive PW stimulation induced a gradual decrease in the spontaneous firing frequency (Figures 2A–C). This inhibitory effect persisted during the post-stimulation period: at 8–10 min after the start of stimulation, the firing frequency was approximately half of the pre-stimulation level. No recovery from the inhibitory effect was observed during the recording period. When we reduced the number of repetitions to 10, the inhibitory effect was attenuated (Figure 2D), indicating the stimulation-dose-dependency of the inhibitory effect. We also tested repeated CW stimulation but found no impact on network activity (Supplementary Figure S6). The inhibitory effect of repeated PW stimulation on neural network activity observed here is consistent with previous findings that this long-lasting pulsed stimulation suppresses epileptic EEG discharges and seizure episodes, suggesting that repetitive ultrasound stimulation acts on either neurons, astrocytes, or both to induce long-lasting neural plasticity.

The balance of activity between excitatory and inhibitory neurons is essential in determining the level of spontaneous network activity in local neural networks of primary cultured neurons (Barral and D’Reyes, 2016). Disruption of this balance in the brain leads to disorders such as epilepsy and schizophrenia (Selten et al., 2018). Decreased neural network activity is likely due to increased inhibitory synaptic input, reduced excitatory synaptic input, or both. We therefore investigated the effects of repetitive PW stimulation when inhibitory synaptic transmission was blocked. When we added the GABA_A_ receptor blocker bicuculline to the bath, the frequency of action potential firing was no longer affected by repetitive PW stimulation (Figures 2E–H). Thus, GABAergic inhibitory synaptic transmission plays a critical role in the inhibitory effects of repetitive ultrasound.

### The inhibitory effect of ultrasound stimulation is due to extrasynaptic, not synaptic, GABA_A_ receptors

Next, we investigated the locus at which repetitive PW stimulation acts on GABAergic synaptic transmission. In neurons, GABA_A_ receptors are found in two distinct locations: synaptic and extrasynaptic. Synaptic receptors are located at the postsynaptic membrane within the synapse and are activated immediately upon binding to GABA, resulting in a transient inhibitory current. This can be observed experimentally as either action-potential-induced IPSCs or as miniature IPSCs (mIPSCs) induced by spontaneous release of GABA from the presynaptic terminal. The frequency of mIPSCs depends on the probability of neurotransmitter release from the presynaptic terminal, and the amplitude depends on the synaptic GABA_A_ receptors. Extrasynaptic GABA_A_ receptors are distributed outside the synapse. These receptors have a high affinity for GABA, are resistant to desensitization, and lead to a sustained increase in membrane conductance. Therefore, a tonic GABA_A_ current is generated in response to the ambient GABA level, which is primarily defined by the rate of GABA release and its uptake by GABA transporters at presynaptic terminals and astrocytes.

**Figure 3:**
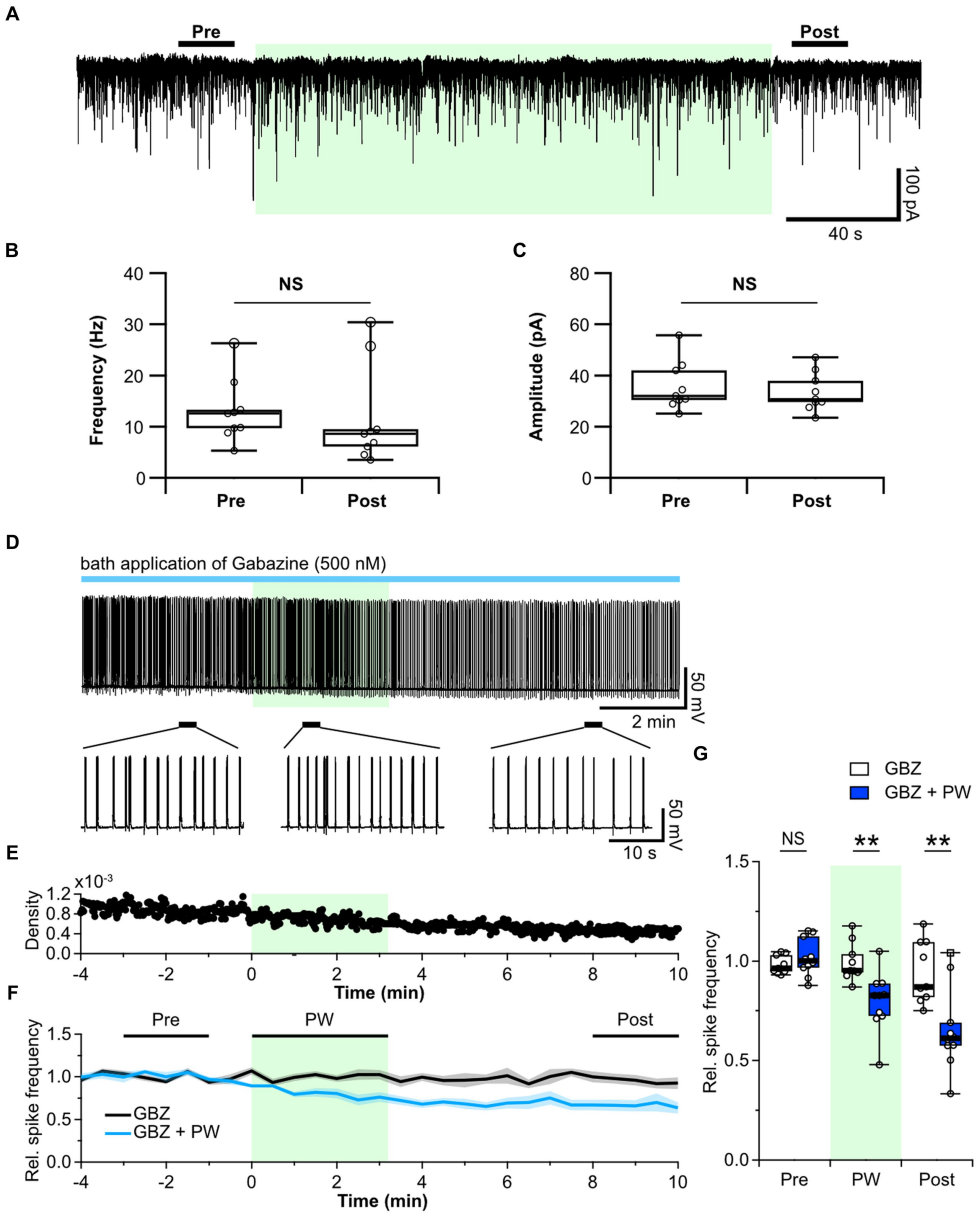
Repetitive PW stimulation affects tonic but not phasic inhibition. **(A)** Representative mIPSC traces before, during and after repetitive PW stimulation. **(B)** Comparison of average event frequency calculated from the 20-s pre and post intervals indicated by the bars in **(A)** (*n* = 9 independent experiments, two-tailed Wilcoxon signed-rank test, *p* = 0.3008). **(C)** Comparison of the average event amplitude calculated from the 20-s pre and post intervals indicated by the bars in **(A)** (*n* = 9 independent experiments, two-tailed Wilcoxon signed-rank test, *p* = 0.09766). **(D)** Representative trace of network activity before, during, and after repetitive PW stimulation in the presence of gabazine (GBZ) (upper). Magnified view of the indicated regions (bottom). **(E)** Spike frequency of the trace in **(D)**. **(F)** Average spike frequency in the pre, PW, and post intervals in the presence of gabazine. **(G)** Comparison of the average spike frequency calculated from the 2-min pre and post intervals and the 3-min PW period, as indicated by the bars in **(F)** (control *n* = 9, gabazine *n* = 10 independent experiments, two-tailed Mann–Whitney *U* test). Light green shading indicates the stimulus period. The mean trace is indicated by a solid line, with the SEM shaded. ^**^p < 0.01. NS, not significant.

We first tested whether repetitive PW stimulation alters synaptic GABAergic transmission and/or the probability of GABA release by measuring the frequency and amplitude of mIPSCs before and after PW stimulation. Ultrasound did not change either property (Figures 3A–C), indicating that repetitive PW does not affect presynaptic GABA release or postsynaptic GABA_A_ receptors.

Next, we used the GABA_A_ receptor antagonist gabazine to investigate whether repetitive PW stimulation alters ambient GABA levels. Whereas bicuculline blocks both synaptic and extrasynaptic GABA_A_ receptors, low concentrations of gabazine have little effect on extrasynaptic GABA_A_ receptors and selectively block only synaptic GABA_A_ receptors (Semyanov et al., 2003; Yamada et al., 2007). We confirmed that 10 μM bicuculline caused a shift in the base current in addition to suppressing phasic GABA_A_ currents (Supplementary Figure S7A). By contrast, administration of 500 nM gabazine selectively suppressed only phasic GABA_A_ currents, with limited suppression of the tonic GABA_A_ current (Supplementary Figures S7B,C). We thus administered gabazine instead of bicuculline to block only the synaptic GABA_A_ receptors and observed the effects of repetitive ultrasound stimulation (Figures 3D–F). Repetitive PW stimulation suppressed network activity even when phasic GABA_A_ currents were blocked by gabazine (Figure 3G), suggesting that the tonic GABA_A_ current is involved in the mechanism of action of ultrasound. In the hippocampus, the primary source of extracellular GABA is vesicular release from nerve terminals (Glykys and Mody, 2007). Since repetitive PW stimulation did not affect the frequency of GABA release from synaptic terminals, we hypothesized that GABA uptake may be affected.

### Involvement of astrocytes in the inhibitory effect of repetitive PW ultrasound

**Figure 4:**
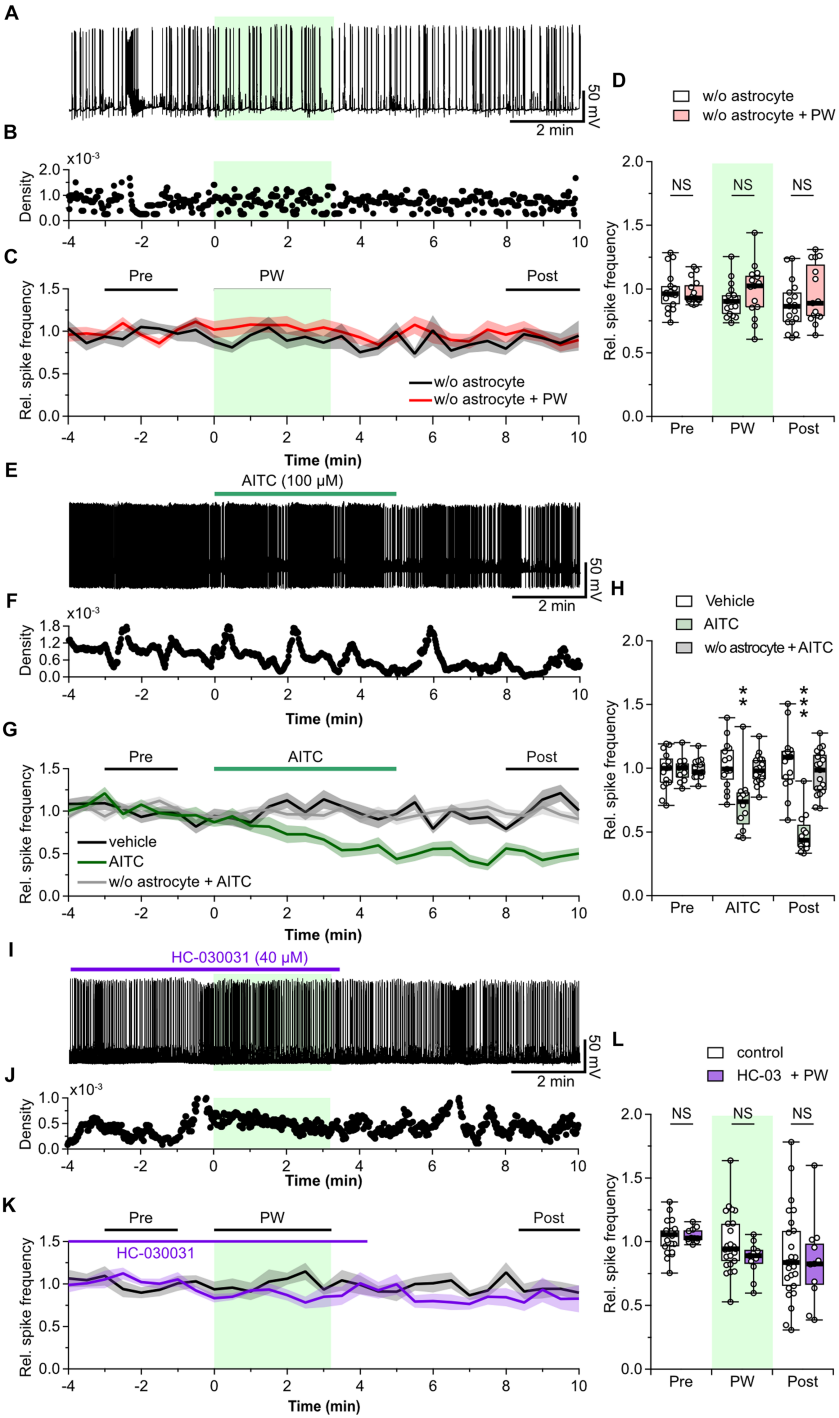
Astrocytes are involved in the neuromodulation by repetitive PW ultrasound. **(A)** Representative trace of network activity before, during, and after repetitive PW stimulation in cultures grown without astrocytes. **(B)** Spike frequency of the trace shown in **(A)**. **(C)** Average spike frequency for the pre, PW, and post intervals. **(D)** Comparison of the average spike frequency calculated from the 2-min pre and post intervals and the 3-min PW period, as indicated by the bars in **(C)** (w/o astrocytes *n* = 17, w/o astrocytes + PW *n* = 15 independent experiments, two-tailed Mann–Whitney *U* test). **(E)** Representative trace of network activity before, during, and after application of AITC. **(F)** Spike frequency of the trace shown in **(E)**. **(G)** Average spike frequency before, during, and after application of AITC. **(H)** Comparison of the average spike frequency calculated from the 2-min pre and post intervals and the 5-min AITC application period, as indicated by the bars in **(G)** (vehicle *n* = 15, AITC *n* = 12, w/o astrocytes + AITC *n* = 19 independent experiments, two-tailed Steel test). **(I)** Representative trace of network activity before, during, and after application of HC-030031. **(J)** Spike frequency of the trace shown in **(I)**. **(K)** Average spike frequency before, during, and after application of HC-030031. **(L)** Comparison of the average spike frequency calculated from the 2-min pre and post intervals and the 3-min PW period, as indicated by the bars in **(K)** (control *n* = 25, HC-030031 *n* = 11 independent experiments, two-tailed Mann–Whitney *U* Test). Light green shading indicates the stimulus period. The mean trace is indicated by a solid line, with the SEM shaded. ^**^p < 0.01, ^***^p < 0.001. NS, not significant.

Uptake of released GABA is mediated by GABA transporters expressed in presynaptic terminals and astrocytes. Therefore, we next investigated whether astrocytes are involved in the mechanism of action of ultrasound. We observed the effect of repetitive PW stimulation on the activity of neurons cultured without an astrocyte feeder layer (Figures 4A–C). In this experiment, ultrasound did not cause a decrease in network activity in the recorded neurons (Figure 4D), indicating that astrocytes are involved in the ultrasound-induced increase in ambient GABA levels. So, how does ultrasound act on astrocytes? ASIC1a (Lim et al., 2021), Piezo1 (Prieto et al., 2018; Chi et al., 2022; Zhu et al., 2023), TRPA1 (Shigetomi et al., 2012; Oh et al., 2019; Duque et al., 2022), and TRPV4 (Shibasaki et al., 2014; Zhuo et al., 2023) are all ultrasound-responsive mechanosensitive receptors expressed in astrocytes. Among these, TRPA1 is expressed in astrocytes (Shigetomi et al., 2013), oligodendrocytes (Hamilton et al., 2016; Giacco et al., 2023), endothelial cells (Thakore et al., 2021), and neurons (Patil et al., 2023), and is more responsive to ultrasound than TRPV4, and Piezo1 (Oh et al., 2019). So, we considered it a strong candidate for involvement in the firing reduction induced by ultrasound stimulation. To investigate whether TRPA1 is involved in the inhibitory effect of repetitive PW stimulation, we pharmacologically activated TRPA1 by bath application of AITC, an agonist of TRPA1, instead of applying ultrasound stimulation (Figures 4E–G). AITC decreased network activity with the same time course as repetitive PW stimulation, and the reduced activity persisted after the application of AITC (Figure 4H). In contrast, AITC did not affect neurons without astrocyte feeder layer (Figures 4G,H). These results suggest that the inhibitory effect induced by ultrasound is mediated by TRPA1 channels on astrocytes. To test this directly, we investigated whether blocking TRPA1 abolishes the inhibitory effect of repetitive PW stimulation (Figures 4I–K). After bath administration of HC-030031, a TRPA1 blocker, we no longer observed an inhibitory effect of repetitive PW stimulation (Figure 4L), indicating that repetitive PW stimulation inhibits network activity in neurons by activating TRPA1 in astrocytes.

**Figure 5:**
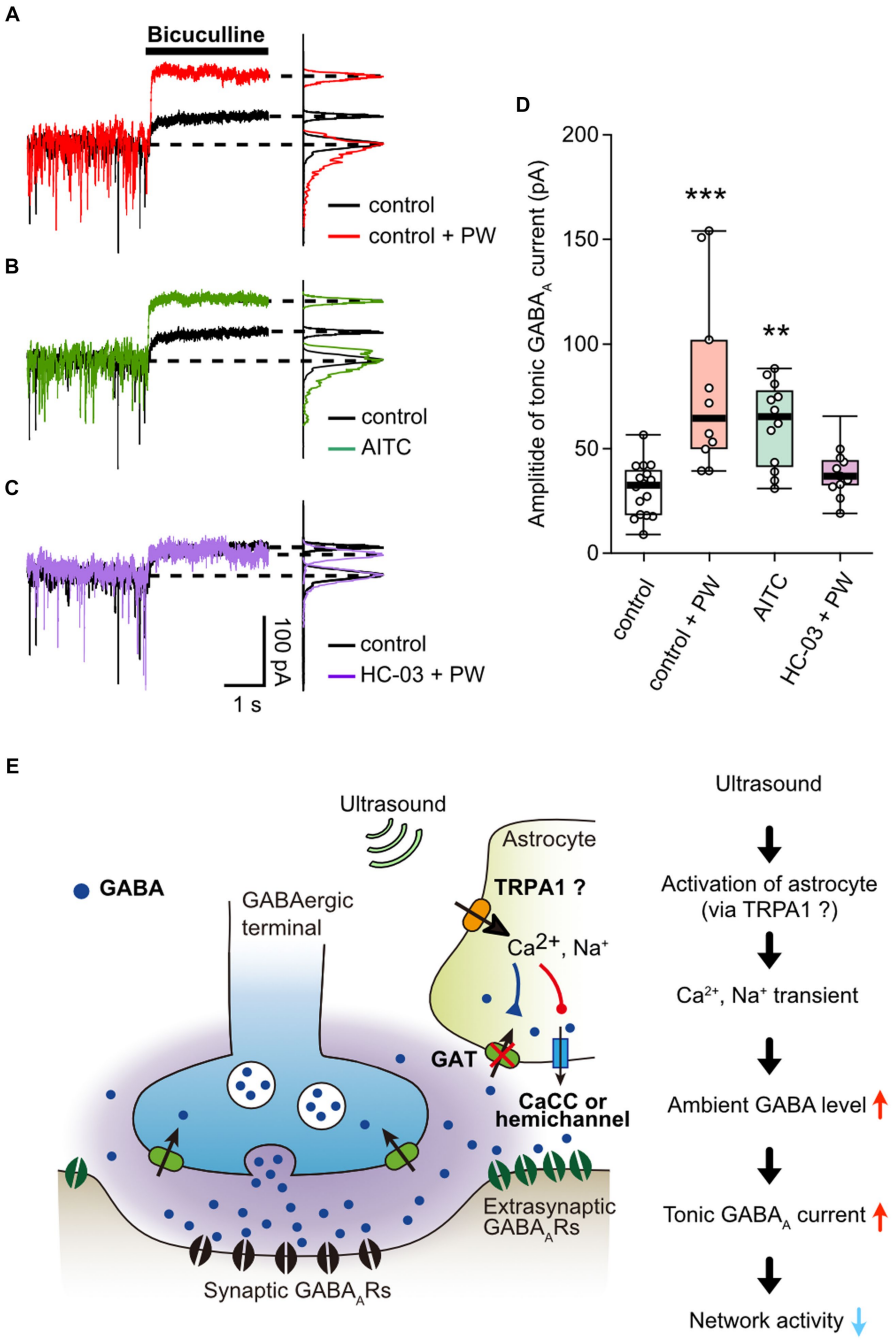
Activation of TRPA1 channels increases ambient GABA levels. **(A)** Representative traces showing the tonic GABA_A_ currents recorded with or without repetitive PW. Bicuculline was applied immediately after the end of the 3-min repetitive PW. The corresponding all-point histograms are shown on the right. **(B)** Representative traces showing the tonic GABA_A_ currents recorded in the presence or absence of AITC. Bicuculline was applied immediately after 5 min of AITC administration. **(C)** Representative traces of the tonic GABA_A_ currents recorded with repetitive PW plus HC-030031. Bicuculline was applied immediately after the end of the 3-min repetitive PW. **(D)** Comparison of the tonic GABA_A_ current amplitude for each condition shown in **(A–C)** (control *n* = 16 independent experiments; control + PW *n* = 10 independent experiments, ^***^p = 0.00067; AITC *n* = 12 independent experiments, ^**^p = 0.00147; HC-030031 + PW *n* = 12 independent experiments, *p* = 0.1476, two-tailed Steel test). **(E)** Proposed model for neuromodulation by repetitive PW stimulation. Ultrasound causes an influx of Ca^2+^ and Na^+^ into astrocytes via channels with characteristics of TRPA1, which leads to (1) decreased uptake of GABA and (2) release of GABA from astrocytes. As a result, ambient GABA levels are elevated, leading to the suppression of neural activity by tonic inhibitory currents. ^**^p < 0.01, ^***^p < 0.001.

Finally, we measured whether activation of TRPA1 by repetitive PW stimulation increases the ambient GABA level. The repetitive ultrasound stimulation increased the amplitude of the tonic GABA current (Figure 5A), in a manner similarly to when TRPA1 channels are activated by AITC (Figure 5B). In addition, pharmacological inhibition of TRPA1 suppressed the effects of repeated ultrasound stimulation on the tonic current (Figure 5C), indicating that repetitive PW increases ambient GABA levels via TRPA1 (Figure 5D).

## Discussion

In this study, we investigated the neuromodulatory effects of different types of patterned ultrasound stimulation on synaptic transmission and network activity in naïve hippocampal neurons using whole-cell recordings. Cells expressing exogenous mechanosensitive channel-expressing cells are often used to analyze the mechanism of action of ultrasound (Kubanek et al., 2016; Yoo et al., 2022; Cadoni et al., 2023) but to apply ultrasound stimulation as a new therapeutic technique for human diseases, it is crucial to confirm the responses of endogenous channels. Therefore, we examined only endogenous channel responses in this study. Until now, calcium imaging has been the predominant tool for analyzing the mechanism of action of ultrasound stimulation (Duque et al., 2022; Yoo et al., 2022; Zhu et al., 2023), and whole-cell recording has rarely been used. Calcium imaging can assess the activation of mechanosensitive channels by measuring intracellular calcium levels. Intracellular Ca^2+^ levels do not, however, sufficiently represent neural function. To study neuronal function, direct measurements of the electrical responses of neurons, such as synaptic currents and neural network activity, are required. Therefore, we used whole-cell recordings to analyze the neural effects of ultrasound stimulation. One factor that has prevented the use of whole-cell recordings in ultrasound experiments is that resonance caused by ultrasound destroys the gigaohm seals. A solution to this is to use frequencies in the MHz range rather than the 500 kHz generally used for stimulation. So far, it has been possible to make recordings with 43 MHz stimulation under intense sound pressure (Prieto et al., 2018, 2020). In addition, recordings with 7 MHz, 0.5 MPa single-shot stimulation have been achieved (Duque et al., 2022). In this study, we integrated an ultrasonic stimulation system into an upright microscope using a small transducer and successfully recorded neural responses to long-term repetitive stimulation at 0.1 MPa and 5 MHz. Achieving successful whole-cell recordings with 5 MHz stimulation is itself a notable feature of this study.

We found that repetitive low-DC low-PRF stimulation (repetitive PW stimulation) activated channels with characteristics of TRPA1 in astrocytes and increased ambient GABA levels, resulting in long-term suppression of neural network activity. Ultrasound stimulation has excitatory and inhibitory effects depending on the species, stimulation site, and parameters (Zhang et al., 2021). Although no clear correspondence between stimulation parameters and effects has been found, stimulation with high DC (30–100%) (Dell’Italia et al., 2022), high PRF (1–2 kHz), and short duration (~400 ms) induced motor responses and neuronal depolarization in the motor cortex (Yuan et al., 2020) and somatosensory cortex (Kim et al., 2014; Li et al., 2019). On the other hand, stimulation with low DC (5–10%) (Dell’Italia et al., 2022), low PRF (100–500 Hz), and long duration (2.5–10 min) continuously suppressed seizures and chemically induced acute epileptic activity in the thalamus (Min et al., 2011), hippocampus (Chen et al., 2020), and visual cortex (Kim et al., 2015). In the present study, long-term (3 min) stimulation at low DC and low PRF had a sustained inhibitory effect on neural network activity in primary cultured cells, suggesting that ultrasound stimulation may be mediated by similar neural mechanisms *in vivo* and *in vitro*. By contrast, CW stimulation, which has been reported to have excitatory effects, had no significant effect on neural activity in our experiments, either with single-shot or repetitive stimulation. Excitatory effects may require intense sound pressure that activates many mechanosensitive channels to induce sufficient depolarization in neurons (Yoo et al., 2022). The conditions for eliciting excitatory effects should continue to be studied.

Recently, it has become clear that calcium-permeable mechanosensitive cation channels are involved in ultrasound reception and the associated transient elevation in intracellular calcium. TRPP1/2, TRPC1, TRPM4, Piezo1 (Yoo et al., 2022), TRPV1 (Yang et al., 2021; Xu et al., 2023), ASIC1a (Lim et al., 2021), TRAAK (Sorum et al., 2021), and TRPA1 (Oh et al., 2019; Dell’Italia et al., 2022) channels are sensitive to ultrasound, but their specific sensitivities vary. ASIC1a (Lim et al., 2021), Piezo1 (Zhu et al., 2023), and TRPA1 (Oh et al., 2019), expressed in astrocytes, respond to low-intensity ultrasound stimulation below 100 kPa. In the present study, our selected patterned ultrasound stimulation did not directly affect neuronal membrane potential or the generation of action potentials. This is consistent with a previous finding that single-shot stimulation at 500 kPa induces a weak inward current insufficient to generate action potentials (Dell’Italia et al., 2022). Another possible site of ultrasound activation is the presynaptic terminal, because ultrasound stimulation induces transient calcium elevations in presynaptic terminals (Tyler et al., 2008). ASIC1a, which is sensitive to low-intensity stimulation, localizes to axons and presynaptic terminals, and is involved in neurotransmitter release (Liu et al., 2020), did not induce exocytosis of synaptic vesicles in response to ultrasound in our study, because the frequency and amplitude of mEPSCs and mIPSCs were not altered. From our results, we conclude that the activation of intrinsic mechanosensitive channels by low-intensity ultrasound stimulation is not large enough to directly activate neurons at 100 kPa sound pressure. Higher-intensity ultrasound stimulation is required to activate endogenous channels and elicit neuronal depolarization (Duque et al., 2022).

Although the ultrasound stimulation used in our study had no direct effect on neuronal function, it had an indirect inhibitory neuromodulatory effect on neurons by increasing ambient GABA levels via channels with characteristics of TRPA1 in astrocytes. The ultrasound-responsive channels expressed in astrocytes include ASIC1a (Huang et al., 2010), Piezo1 (Velasco-Estevez et al., 2018), TRPV1 (Tóth et al., 2005), TRPV4 (Shibasaki et al., 2014), and TRPA1 (Oh et al., 2019; Duque et al., 2022). Of these, TRPA1 is more responsive to ultrasound than TRPC1, TRPV4, and Piezo1 (Oh et al., 2019), and activated at lower sound pressure than TRPV1 and TRPC1 (Duque et al., 2022), which supports our results. The involvement of TRPA1 as a starting point for the neuromodulatory effects of low-intensity ultrasound is consistent with a previous report by Oh et al. (2019), but the direction of the effect we observed was the opposite. Oh et al. (2019) reported that the ultrasound-induced transient calcium influx via TRPA1 channels activates a calcium-activated chloride channel (CaCC), bestrophin 1 (BEST1) (Park et al., 2009), which releases glutamate as a gliotransmitter and excites neurons. In addition to BEST1, astrocytes express CaCCs such as LRRC8A (swell 1) (Formaggio et al., 2019), which release glutamate and GABA as gliotransmitters. Our results indicated that ultrasound induced neither depolarization nor action potentials in the recorded neurons. On the contrary, activation of TRPA1 reduced network activity and increased the tonic GABA_A_ current, indicating that astrocytes released GABA but not glutamate or that GABA uptake was reduced (Cheng et al., 2023). Since the release of gliotransmitters through CaCCs depends on the amplitude of the calcium transient, the membrane potential, and the concentration difference of each gliotransmitter across the membrane (Lee et al., 2010), the experimental conditions (organotypic slice culture vs. neuron–astrocyte co-culture), stimulus pattern (DC 2.4%, PRF 1.16 kHz vs. DC 5%, PRF 100 Hz), and sound pressure (11.5 kPa vs. 100 kPa) may underlie the differences in the effects observed. Astrocytes in culture exhibit characteristics of reactive astrocytes (Li et al., 2018), which may promote GABA synthesis and release (Chun et al., 2018). In socially isolated mice, increased TRPA1 expression and GABA synthesis enhanced GABA release from astrocytes and increased the extracellular GABA concentration (Cheng et al., 2023), consistent with our results. In addition, gap junction hemichannels may be another pathway through which GABA is released from astrocytes (Ye et al., 2003). This pathway regulates tonic neuronal GABA_A_ currents in cultured hippocampal neurons and slices (Ransom et al., 2017). The neuron-astrocyte co-culture system used in this study has the limitation that extracellular GABA levels and astrocyte status are different from those in slices and *in vivo*, further studies are needed to analyze the mechanism of gliotransmitter release by ultrasound stimulation.

Because the primary source of extracellular GABA in the normal hippocampus is release from synaptic terminals (Glykys and Mody, 2007), the fact that repeated ultrasound stimulation does not affect the frequency of mIPSCs (and thus does not affect GABA release) suggests that the ambient GABA level increment is due either to decreased GABA uptake or release of GABA from astrocytes. In astrocytes, GAT-1 and GAT-3 function to regulate GABA uptake (Ghirardini et al., 2018). Activation of TRPA1 induces a Na^+^ transient, which reduces GABA uptake by decreasing the driving force of the GABA transporter (GAT) (Jensen et al., 2003). In addition, transient Ca^2+^ influx activates the Na^+^/Ca^2+^ exchanger and produces a further elevation of intracellular Na^+^, thereby reducing GABA uptake (Hilge, 2012). Furthermore, depending on the intracellular GABA and Na^+^ concentrations, the GABA transporter can cause GABA release if it operates in the reverse mode (Héja et al., 2009). In flies, elevated calcium in astrocytes causes GAT endocytosis and reduced functional expression of GAT, resulting in elevated extracellular GABA levels (Zhang et al., 2017). In our study, repetitive PW stimulation inhibited network activity by increasing the tonic GABA_A_ current but not phasic GABA_A_ currents. The inhibitory effect depended on the number of PW repetitions and persisted for more than 10 min after the onset of stimulation. This indicates a continuous elevation in the ambient GABA level due to plastic changes in GABA uptake, release, and synthesis capacity, caused by ultrasound-induced and TRPA1-mediated calcium and sodium signaling in astrocytes (Figure 5E). In our experiments, 100 kPa sound pressure was insufficient to cause direct changes in the membrane potential in neurons but was strong enough to activate channels with characteristics of TRPA1 and induce these plastic changes.

The present results indicate that one of the specific patterns of ultrasound stimulation we examined in this study activated channels with characteristics of TRPA1 in astrocytes to modulate extracellular GABA levels. TRPA1 regulates calcium dynamics in pathological reactive astrocytes (Bosson et al., 2017) and normal astrocytes (Shigetomi et al., 2012) and modulates neuronal function. Activation of TRPA1 channels by ultrasound could be a new non-invasive neuromodulatory method for manipulating neuronal function in local brain regions. To apply this technology to the treatment of neurological diseases, further investigation of the molecular mechanisms by which TRPA1 activation modulates ambient GABA levels and verification of the effects *in vivo* are needed.

## Data availability statement

The raw data supporting the conclusions of this article will be made available by the authors, without undue reservation.

## Ethics statement

The animal study was approved by The Committee for Laboratory Animal Care and Use at Kyorin University (Reference number 222). The study was conducted in accordance with the local legislation and institutional requirements.

## Author contributions

TM: Writing – original draft, Writing – review & editing. KK: Data curation, Writing – review & editing. MT: Data curation, Writing – review & editing. TK: Data curation, Writing – review & editing. AS: Data curation, Writing – review & editing. YU: Writing – review & editing. YT: Writing – review & editing.

## Supplementary material

The Supplementary material for this article can be found online at:

https://www.frontiersin.org/articles/10.3389/fncel.2024.1361242/full#supplementary-material
