# Supplementary figures and images for: Repetitive pulsed-wave ultrasound stimulation suppresses neural activity by modulating ambient GABA levels via effects on astrocytes
Source: Front Cell Neurosci. 2024 Mar 27;18:1361242. doi: 10.3389/fncel.2024.1361242 (PMC11004293; doi:10.3389/fncel.2024.1361242)

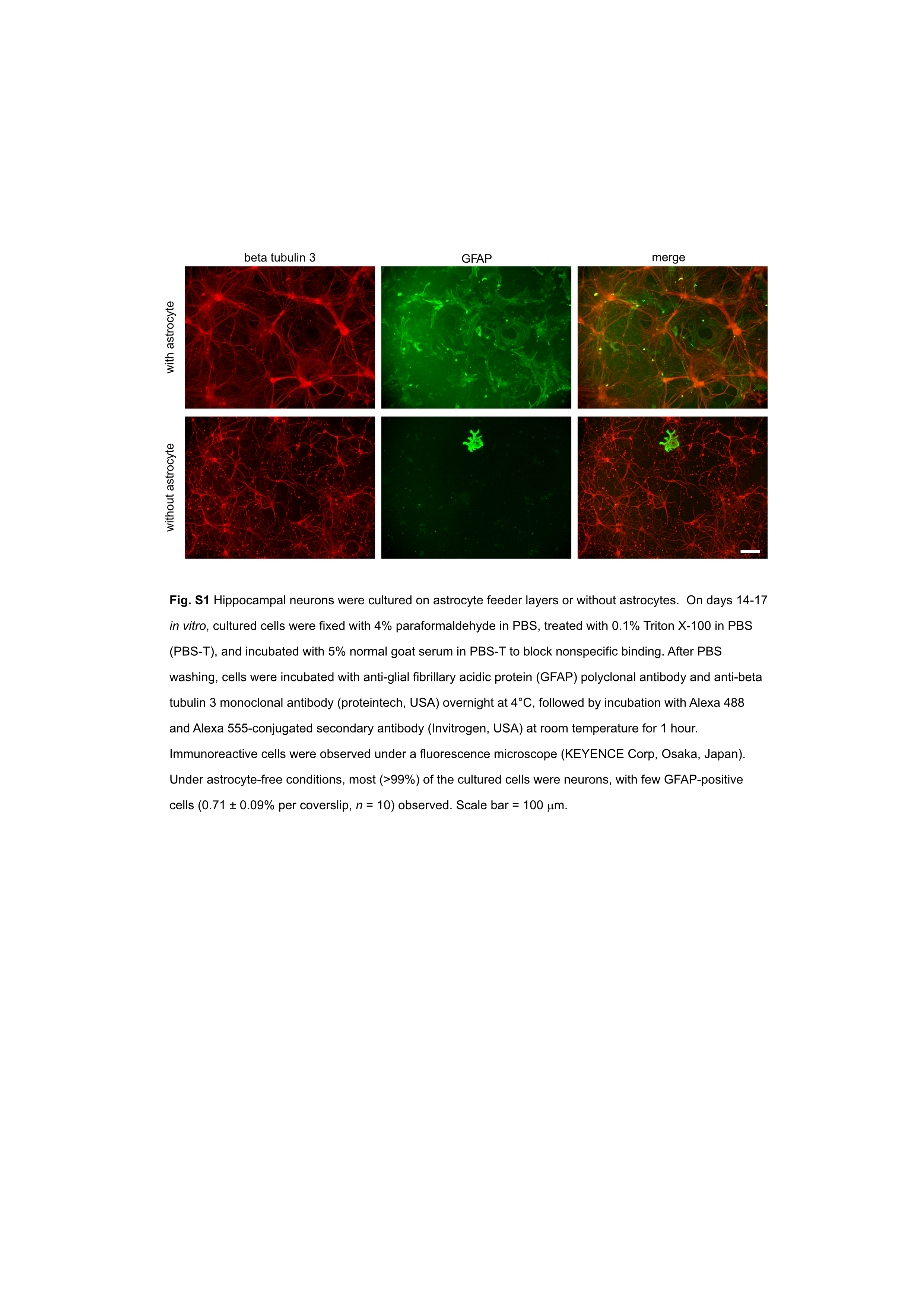

Supplement: Supplementary file 1 [file Image_1.tiff]

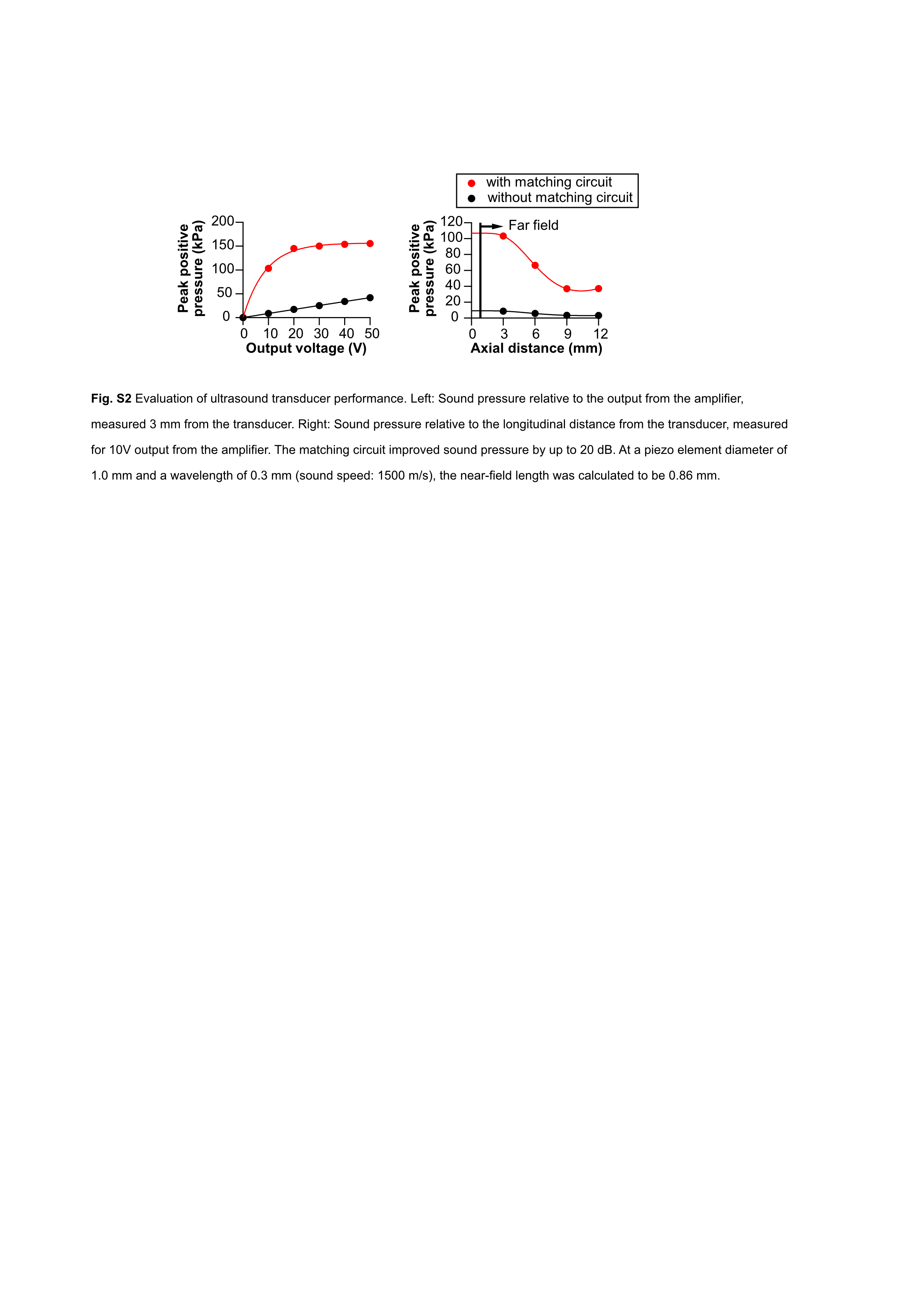

Supplement: Supplementary file 2 [file Image_2.TIFF]

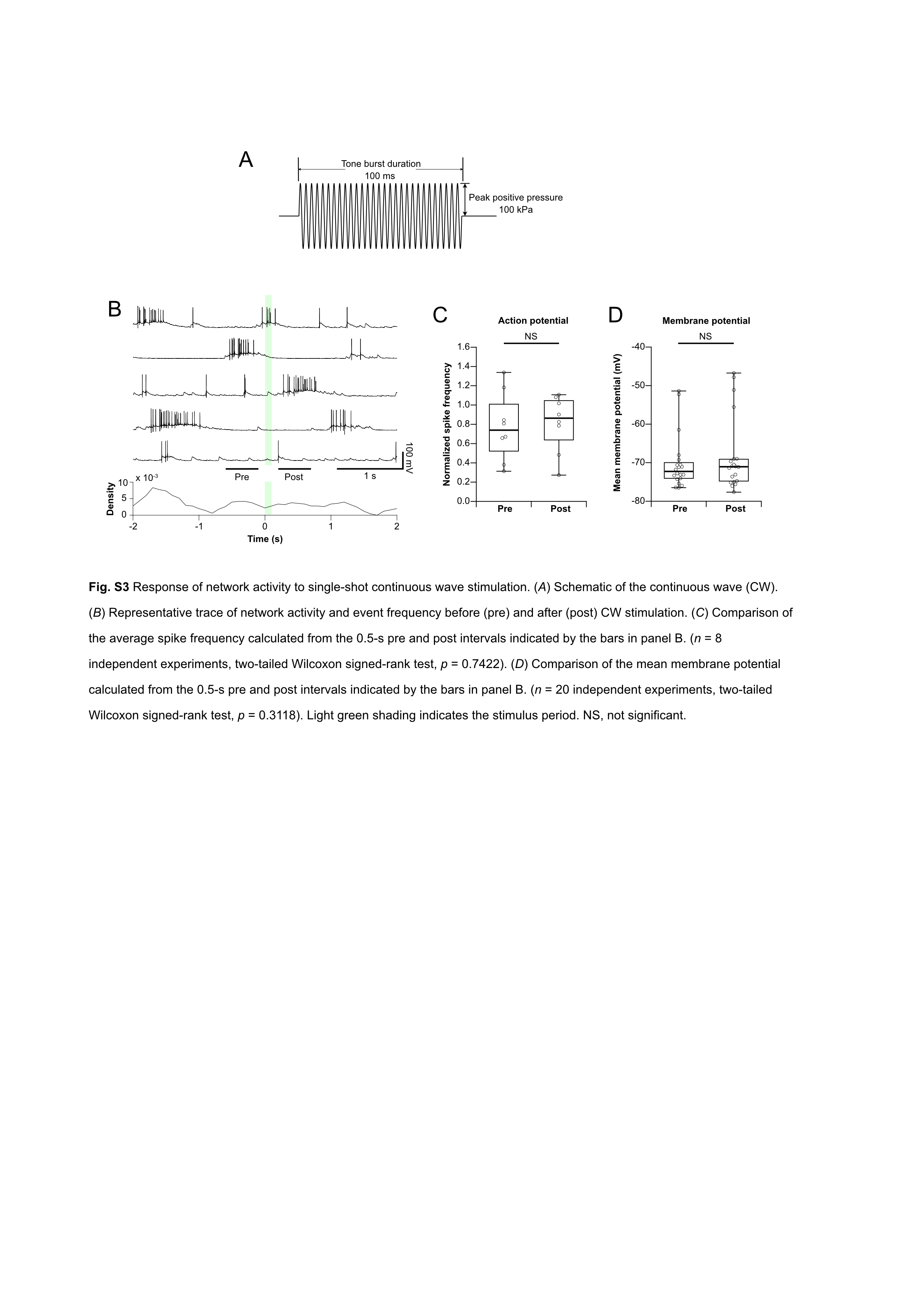

Supplement: Supplementary file 3 [file Image_3.TIFF]

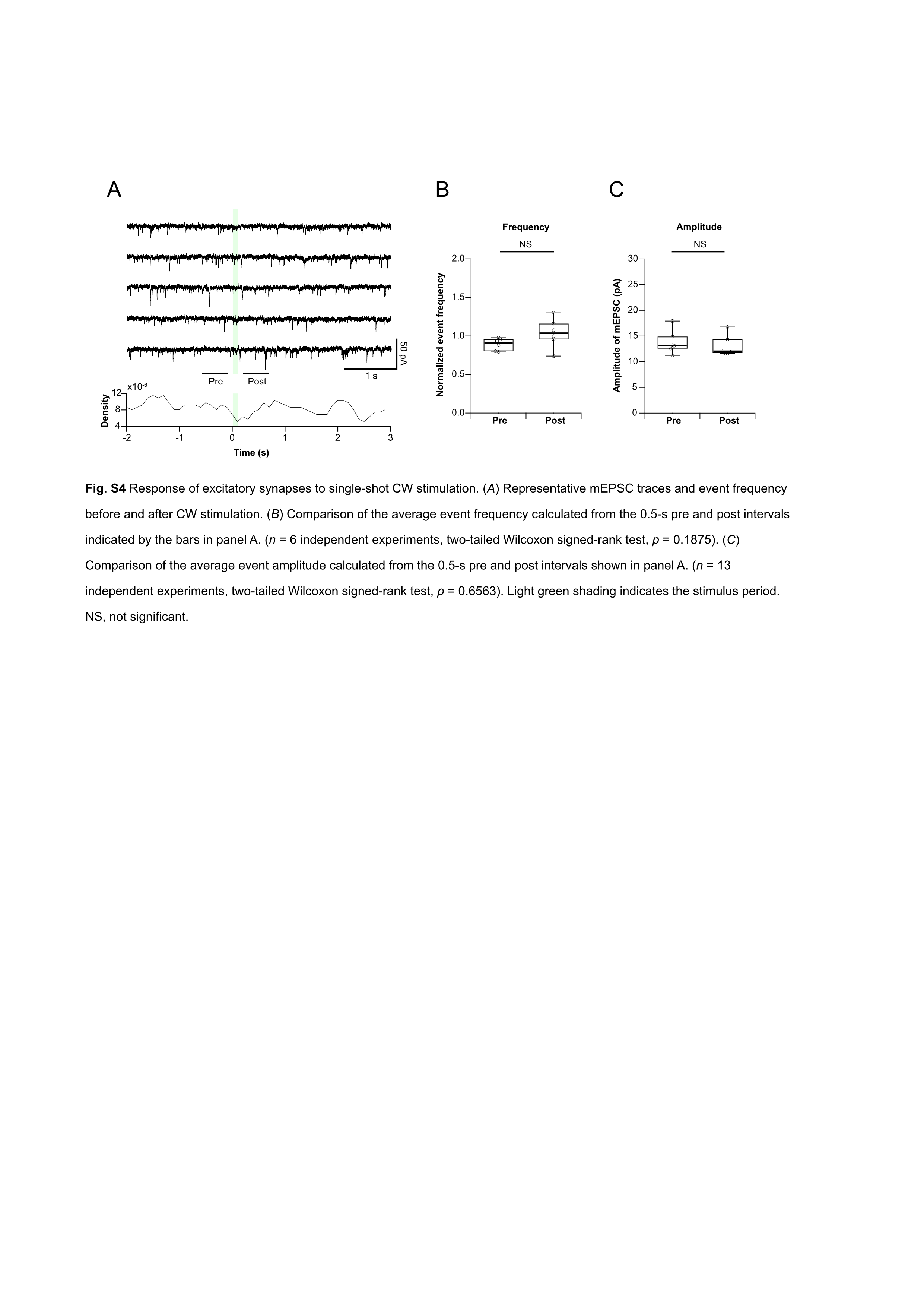

Supplement: Supplementary file 4 [file Image_4.TIFF]

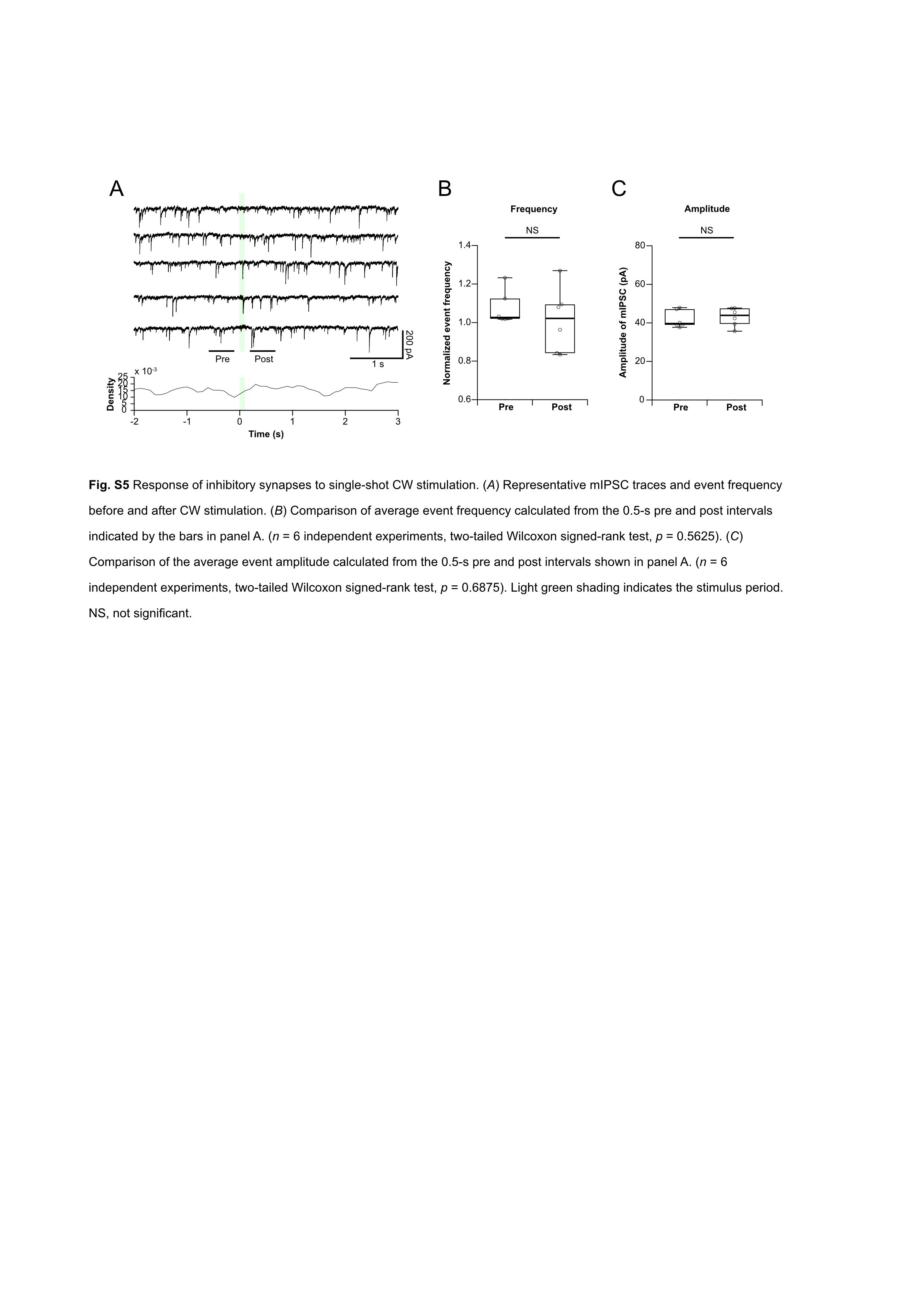

Supplement: Supplementary file 5 [file Image_5.TIFF]

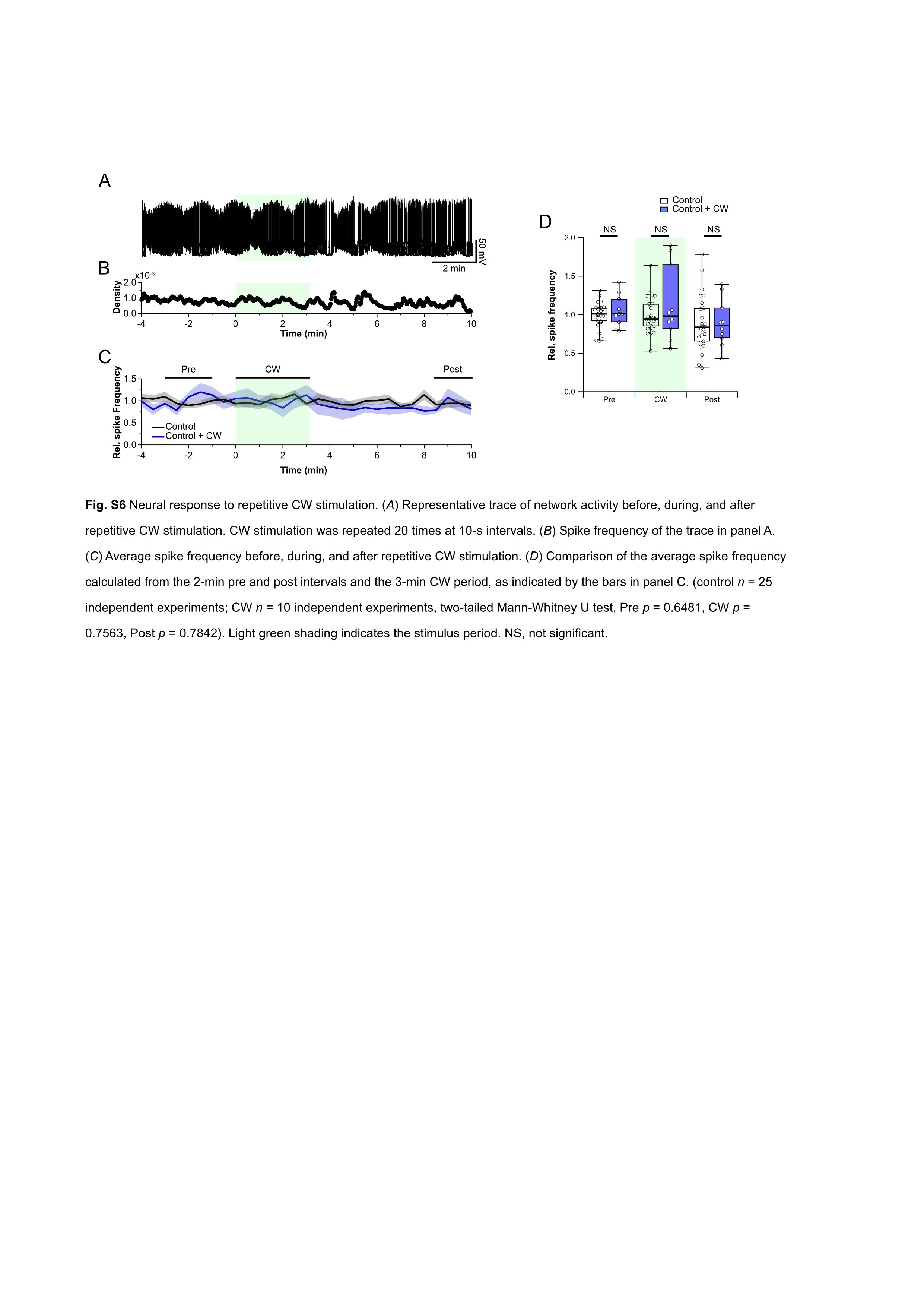

Supplement: Supplementary file 6 [file Image_6.TIFF]

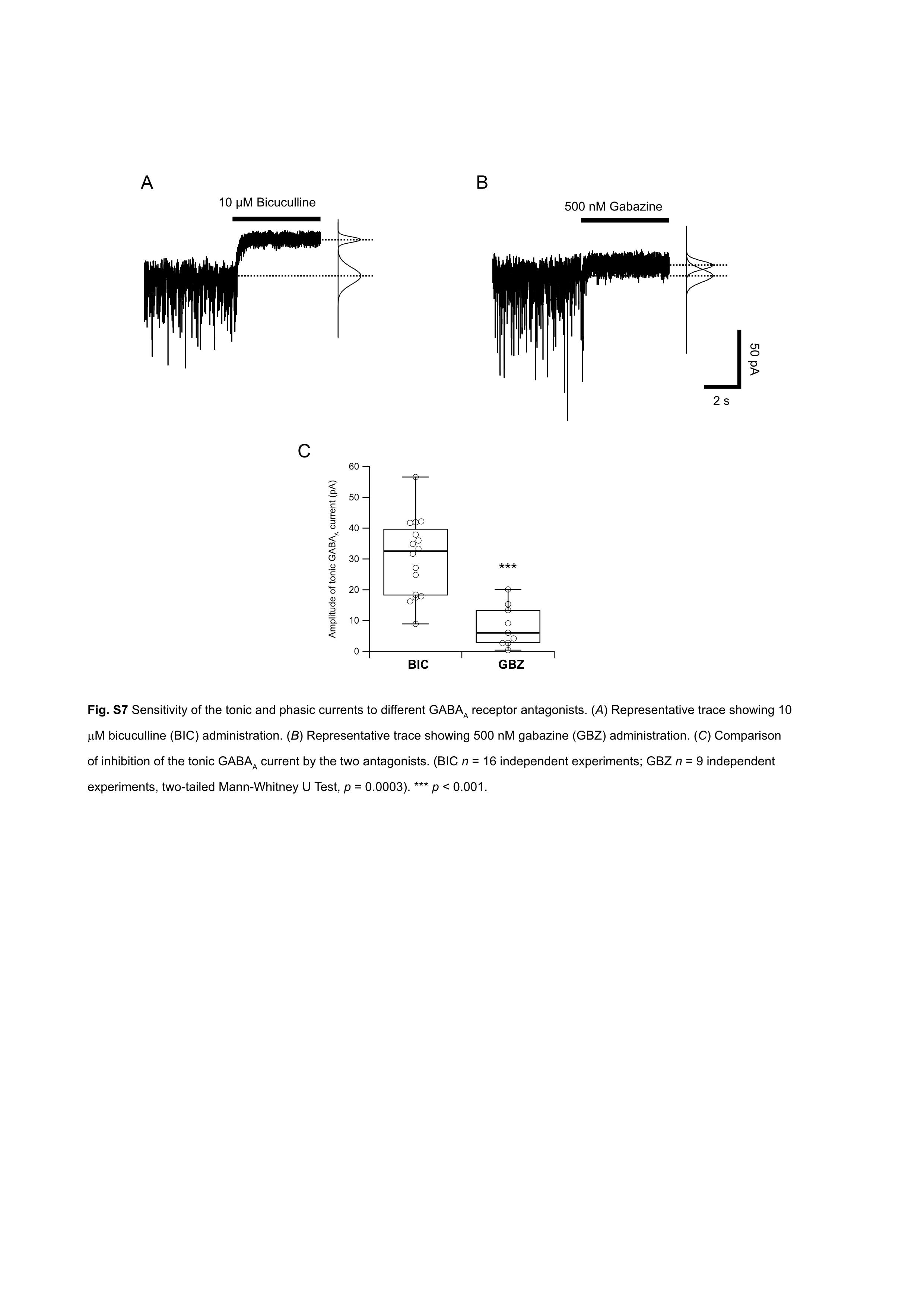

Supplement: Supplementary file 7 [file Image_7.TIFF]
